# Correct dosing of artemether-lumefantrine for management of uncomplicated malaria in rural Tanzania: do facility and patient characteristics matter?

**DOI:** 10.1186/1475-2875-12-446

**Published:** 2013-12-10

**Authors:** Irene M Masanja, Majige Selemani, Rashid A Khatib, Baraka Amuri, Irene Kuepfer, Dan Kajungu, Don de Savigny, S Patrick Kachur, Jacek Skarbinski

**Affiliations:** 1 Ifakara Health Institute, PO Box 78373, Dar es Salaam, Tanzania; 2 Swiss Tropical and Public Health Institute, Socinstrasse 57, CH-4002, Basel, Switzerland; 3 University of Basel, Petersplatz 1, CH-4003, Basel, Switzerland; 4 INDEPTH Network Effectiveness and Safety Studies of Antimalarial Drugs in Africa, Dar es Salaam, Tanzania; 5 Malaria Branch, Center for Global Health, US Centers for Disease Control and Prevention, Atlanta, USA

**Keywords:** Artemether lumefantrine dosing, Uncomplicated malaria, Tanzania

## Abstract

**Background:**

Use of artemisinin-based combination therapy (ACT), such as artemether-lumefantrine (AL), requires a strict dosing schedule that follows the drugs’ pharmacokinetic properties. The quality of malaria case management was assessed in two areas in rural Tanzania, to ascertain patient characteristics and facility-specific factors that influence correct dosing of AL for management of uncomplicated malaria.

**Methods:**

Exit interviews were conducted with patients attending health facilities for initial illness consultation. Information about health workers’ training and supervision visits was collected. Health facilities were inventoried for capacity and availability of medical products related to care of malaria patients. The outcome was correct dosing of AL based on age and weight. Logistic regression was used to assess health facility factors and patient characteristics associated with correct dosing of AL by age and weight.

**Results:**

A total of 1,531 patients were interviewed, but 60 pregnant women were excluded from the analysis. Only 503 (34.2%) patients who received AL were assessed for correct dosing. Most patients who received AL (85.3%) were seen in public health facilities, 75.7% in a dispensary and 91.1% in a facility that had AL in stock on the survey day. Overall, 92.1% (463) of AL prescriptions were correct by age or weight; but 85.7% of patients received correct dosing by weight alone and 78.5% received correct dosing by age alone. In multivariate analysis, patients in the middle dosing bands in terms of age or weight, had statistically significant lower odds of correct AL dosing (p < 0.05) compared to those in the lowest age or weight group. Other factors such as health worker supervision and training on ACT did not improve the odds of correct AL dosing.

**Conclusion:**

Although malaria treatment guidelines indicate AL dosing can be prescribed based on age or weight of the patient, findings from this study show that patients within the middle age and weight dosing bands were least likely to receive a correct dose by either measure. Clinicians should be made aware of AL dosing errors for patients aged three to 12 years and advised to use weight-based prescriptions whenever possible.

## Background

The World Health Organization (WHO) declared that much of ill health, disease, premature death, and suffering are needless, since efficacious and affordable interventions for prevention and treatment are available. It is further noted that these effective interventions are not matched by the strength of health systems to deliver them to those in greatest need in a comprehensive way and on an adequate scale [1]. This fact highlights the need to assess and address health systems’ bottlenecks, in order to improve health outcomes and the quality of health care services.

Following widespread resistance by malaria parasites to commonly used anti-malarials, such as chloroquine and sulphadoxine-pyrimethamine (SP), there was a global move to use artemisinin-based combination therapy (ACT) for malaria treatment [2]. This required the artemisinin derivatives with shorter half-life be combined with a longer half-life partner drug, to enhance therapeutic efficacy and reduce treatment durations [2]. Currently, the artemisinin derivatives used for malaria combination treatment include artesunate, artemether, and dihydroartemisinin (DHA). ACT, such as artemether-lumefantrine (AL), became first-line medicine for management of uncomplicated malaria in many malaria-endemic countries, including Tanzania [3].

Good quality malaria case management entails that all true malaria cases be appropriately identified, and that all identified cases be treated with efficacious anti-malarial medicines. To achieve desired treatment outcomes, sufficient blood levels of the active medical ingredient must be reached. The increased use of microscopy and malaria rapid tests for parasitological confirmation of malaria improves identification of true malaria patients. Despite the limitations of malaria rapid tests, these tests have been introduced in routine care and have resulted in better targeting of anti-malarials in endemic countries [4,5]. Previous studies in malaria endemic countries in Sub-Saharan Africa have documented factors affecting quality malaria case management. Challenges related to under-use of malaria testing, distrust of negative results, ambiguous treatment policies and un-availability of recommended medicines are contributing factors [6,7].

Poor adherence and inappropriate use of recommended treatment have been linked to the development and spread of drug resistance [8,9]. Use of medicines requires a strict dosing schedule that aligns with the drug’s pharmacokinetics [10]. The current dosing of AL is based on four predefined weight bands and age groups. According to manufacturers, an eight-hour interval between the first and the second doses of AL is critical for appropriate parasite clearance and clinical cure. In addition, manufacturers recommend completing the doses at defined time intervals and taking AL with a fatty meal for better absorption of the drug to enhance its bioavailability. These recommendations, if followed thoroughly, will optimize therapeutic response. Unfortunately many studies have reported that clinical practice differs for various reasons [7,11,12].

Packaging of AL medicines for public sector use in Tanzania is customized for each dosing band and incorporates illustrated instructions for patients and caretakers with low literacy. Both age-based or weight-based AL dosing recommendations are included in the guidelines [3], and therefore it is at service provider’s discretion to prescribe based on either of the two dosing methods. This study was carried out to assess the quality of malaria case management, including an assessment of AL dosing, in a real world setting. Factors influencing correct AL dosing were explored to inform disease control programme and local health management teams to plan effective malaria-related supportive supervision, focusing on areas that require emphasis.

## Methods

### Study design and study area

Data were collected through a pair of cross-sectional health facility surveys in the Rufiji and Ifakara Health and Demographic Surveillance System (HDSS) areas, in March and October 2010. All health facilities, public and private, within the HDSS areas were included in the survey; 16 health facilities from Rufiji HDSS and 14 health facilities from Ifakara HDSS, as previously described [5]. This work was completed as part of the large phase 4 platform of the INDEPTH Network to assess effectiveness and safety of anti-malarial drugs called INESS- Indepth Network Effectiveness and Safety Studies of anti-malarial drugs [13].

### Data collection

All outpatients presenting for initial illness consultation on the day of a survey were approached to assess eligibility and seek consent for inclusion in the study. Information was collected by means of paper questionnaires and through interviews with patients or caretakers. Information on patients’ complaints, provider’s diagnosis, dosing instructions, and counselling messages were recorded. Patients’ clinical notes were later reviewed for similar information to ascertain concordance between patients’ narration and clinical notes. Information from clinical notes was also recorded in the questionnaire. Patients were weighed and asked for a blood smear for independent assessment of presence of malaria parasites. Results of microscopic analysis of blood smears were not available immediately for clinical judgement; but were sent back to the facility after two or three days. Information on provider’s training, work experience and supervision visits were also recorded. The facilities were inventoried for availability of medicines, diagnostics, and reference materials related to malaria treatment.

### Data entry and analysis

Data were entered in EPIDATA Entry version 3.1 (EpiData Association, Odense, Denmark) by two independent entry clerks. Analysis was performed in STATA 11 (StataCorp, College Station, Texas, USA). Descriptive analysis was undertaken after merging health facility, health worker and patient datasets. Pearson Chi-squared test was used to compare proportions of patients who received correct AL dose based on age and/or weight by different patients’ characteristics and facility factors. Logistic regression was carried out to assess association of health facility factors and patients’ characteristics with correct AL dosing. Health facility factors assessed were type of facility, supervision within the past six months, training on AL, availability of reference materials on treatment guideline as well as presence of AL, diagnostics, and weighing scales. Patients’ characteristics assessed were age, weight, fever or history of fever in the previous 24 hours, laboratory tests performed, and treatment provided. All predictors of correct dosing were run simultaneously in the adjusted model. An alpha level of 0.05 was used for all significance tests. All analyses accounted for the complex sample survey design of this study and accounted for clustering with the cluster defined as all consultations conducted in a health facility in one day.

### Definition of correct dosing of artemether-lumefantrine

A composite measure was developed to assess correct dosing of AL based on two criteria: i) appropriate number of AL tablets per dose given based on patient’s age or body weight as per national malaria treatment guideline [3], and, ii) appropriate number of doses, i.e., two doses per day for three days. Any dosing instruction that did not meet these criteria was considered inappropriate.

### Ethical clearance

This work was granted ethical permit from the Ifakara Health Institute (IHI/IRB/No. A 67–2009) and the National Institute for Medical Research in Tanzania (NIMR/HQ/R.8a/Vol.IX/871).

## Results

**Figure 1:**
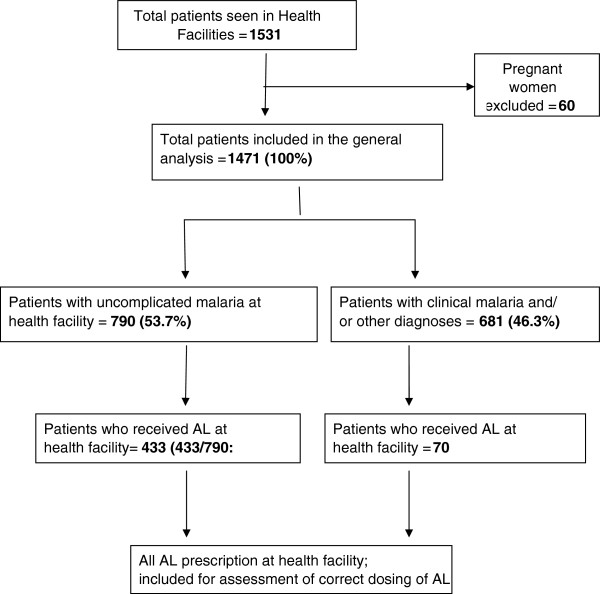
Distribution of study participants.

Of the 1,531 patients who were interviewed, only 503 (32.8%) who received AL were included in the analysis (Figure 1). Table 1 shows the baseline characteristics of patients included in the larger study of case management quality in the HDSS areas. The majority of patients included in the dosing analysis were seen in dispensaries 381 (75.7%) and public health facilities 429 (85.3%) (Table 2). Most patients who were prescribed with AL were seen in a facility that had AL in stock on the day of a survey 458 (91.1%), but only 55.3% (278) patients were seen in a facility with malaria diagnostic test available on the survey day. Although 367 (72.9%) patients were seen in a facility that had a functioning weighing scale, only a third 170 (33.8%) had their weight measured and recorded during a provider-patient interaction, but almost all patients 482 (95.8%) had their age assessed and recorded during clinical consultation. Very few patients were seen by a health worker who had had a supervision visit in the previous six months 110 (21.8%) or who had training on AL use 224 (44.5%). Most patients reported fever or history of fever in the previous 24 hours 488 (97.2%) but only about half of them 223 (44.3%) were sent for malaria testing at the facility.

**Table 1:** Baseline characteristics of all patients included in the survey, by HDSS site

**Facility/patients characteristic**	**All sites: N = 1471(%)**	**Rufiji: n = 710 (%)**	**K/U: n = 761 (%)**
**Type of health facility:**			
Seen in dispensaries	1037 (70.5)	477 (67.2)	560 (73.6)
Seen in health centers	403 (27.4)	202 (28.5)	201 (26.4)
Seen in hospitals	31 (2.1)	31 (4.4)	0
**Health facility ownership:**			
Seen in Public HFs	1179 (80.0)	543 76.5)	636 (83.6)
Seen in Non-public HFs	292 (20.0)	167 (23.5)	125 (16.4)
**Availability:**			
Seen in HF with functioning scale for all age	1428 (97.0)	697 (98.2)	731 (96.1)
Seen in HF with malaria diagnostic capacity	881 (60.0)	527 (74.2)	354 (46.5)
Seen in HF AL in stock	1268 (86.2)	666 (93.8)	602 (79.1)
Seen in HF with treatment reference materials	1293 (87.9)	604 (85.1)	689 (94.4)
**Health worker (HW) factors:**			
Seen by HW trained on AL use	837 (57.0)	219 (30.9)	618 (81.2)
Seen by HW supervised in past 6 months	1004 (68.3)	474 (66.8)	530 (75.6)
**Patients characteristics:**			
Age <5 years	701 (47.7)	349 (49.2)	352 (46.3)
Age 5–15 years	246 (16.7)	112 (15.8)	134 (18.0)
Age >15 years	524 (35.6)	249 (35.1)	275 (36.0)
Fever or history of fever	1247 (84.8)	620 (87.3)	627 (82.4)

**Table 2:** Distribution of patients who received artemether-lumefantrine by health facility, health worker and patient characteristics (N = 503)

**Patient seen in (type of) health facility:**	**n (%)**
Dispensary	381 (75.7)
Health centre	114 (22.7)
Hospital	8 (1.6)
Public health facility	429 (85.3)
Non-public health facility	74 (14.7)
**Availability at health facility:**	
Artemether-lumefantrine in stock	458 (91.1)
Diagnostic capacity rapid diagnostic test or functional microscopy available	278 (55.3)
Weighing scale available	367 (72.9)
Printed copy of malaria treatment guidelines or reference available	411 (81.7)
**Patient seen by health worker who had:**	
Supervision in previous six months	110 (21.8)
Training on use of artemether-lumefantrine	224 (44.5)
**Patient age groups:**	
Aged <3 years	177 (35.2)
Aged 3 to <9 years	144 (28.6)
Aged 9 to <12 years	33 (6.6)
Aged 12 years and above	149 (29.6)
**Patient weight groups:**	
Weight <15 kg	245 (48.7)
Weight 15 to <25 kg	89 (17.7)
Weight 25 to <35 kg	24 (4.8)
Weight 35 kg and above	145 (28.8)
**Other characteristics:**	
Presented with fever/history of fever in previous 48 hours	488 (97.0)
Had weight assessed/recorded	170 (33.8)
Had age assessed/recorded	482 (95.8)
Had fever and a malaria test performed at health facility	223 (44.3)

In all, (463) 92.1% of patients who were prescribed AL received correct dosing by weight and/or age, as indicated in national treatment guidelines (Table 3), and (431) 85.7% of patients received correct dosing based on weight alone and (395) 78.5% based on age alone. Assessment of patients’ characteristics (age and weight) indicated that the proportion of patients who received correct AL dosing was significantly lower in patients within the middle weight bands, i.e., between 15 and 25 kg and 25 and 35 kg, and middle age bands, three to nine years and nine to 12 years (p < 0.001).

**Table 3:** Proportion of patients who received correct dosing of artemether-lumefantrine based on age and/or weight, by health facility, health worker and patient characteristics

**Patient seen in health facility**	**n (%)**	**p- value**
Dispensary (N = 381)	350 (91.8)	0.827
Health centre (N = 114)	106 (93.0)	
Hospital (N = 8)	7 (87. 5)	
Public health facility (N = 429)	398 (92.7)	0.147
Non-public health facility (N = 74)	65 (87.8)	
**Availability at health facility:**		
Artemether-lumefantrine in stock (N = 458)	422 (92.1)	0.808
Diagnostic capacity (mRDT or functional BS:N = 278)	256 (92.1)	0.972
Weighing scale available (N = 367)	340 (92.6)	0.418
Copy of malaria treatment guidelines or reference (N = 411)	379 (92.2)	0.771
**Patient seen by health worker who had:**		
Supervision in previous six months (N = 110)	100 (90.9)	0.618
Training on use of artemether-lumefantrine (N = 224)	203 (90.6)	0.291
**Patient age:**		
Aged <3 years (N = 177)	172 (97.2)	<0.001
Aged 3 to <9 years (N = 145)	123 (84.8)	
Aged 9 to <12 years (N = 33)	27 (81.8)	
Aged 12 years and above (N = 148)	141 (95.3)	
**Patient weight:**		
5 -15 kg (N = 241)	234 (97.1)	<0.001
>15 -25 kg (N = 88)	70 (79.5)	
>25- 35 kg (N = 24)	18 (75.0)	
>35 kg (N = 150)	141 (94.0)	
**Other characteristics:**		
Had fever with present illness (N = 488)	449 (92.0)	0.852
Correct dosing by age or/and weight (N = 503)	463 (92.1)	
Correct dosing by weight only (N = 503)	431 (85.7)	
Correct dosing by age only (N = 503)	395 (78.5)	

In a multivariate analysis, the most important factor for incorrect dosing was patients aged between three and 12 years and body weight >15 to 35 kg compared to children below age three years and <15 kg, respectively (Table 4). Private providers (non-public facilities) also had lower odds of correct dosing in the adjusted analysis (adjusted odds ratio (aOR) 95% confidence interval (CI)): 0.2 (0.06-0.6)). Other factors such as availability of reference materials, supervision, training, availability of AL, and weighing scales at facility were not associated with correct AL dosing (Table 3).

**Table 4:** Predictors of correct dosing of artemether-lumefantrine by age and/or weight

**Patient seen in health facility (N)**	**n (%)**	**Crude OR (95% CI)**	**Adjusted OR (95% CI)**
Dispensary (N = 381)	350 (91.8)	1.6 (0.1-19.6)	2.5 (0.2-26)
Health centre (N = 114)	106 (93.0)	1.8 (0.1-16.3)	1.7 (0.2-17)
Hospital (N = 8)	7 (87.5)	Ref	Ref
Public health facility (N = 429)	398 (92.8)	Ref	Ref
Non-public health facility (N = 74)	65 (87.8)	0.5 (0.2-1.2)	0.2 (0.06-0.6)
**Availability at health facility:**			
Artemether-lumefantrine in stock (N = 458)	422 (92.1)	1.1 (0.4-2.9)	0.9 (0.4-2.1)
No artemether-lumefantrine in stock (N = 45)	41 (91.1)	Ref	Ref
Diagnostic capacity: mRDT or functional BS (N = 278)	256 (92.1)	1.0 (0.5-2.0)	1.8 (0.5-6.1)
No diagnostic capacity (N = 225)	207 (92.0)	Ref	Ref
Weighing scale available (N = 367)	340 (92.6)	0.7 (0.3-1.5)	0.6 (0.3-1.5)
No weighing scale available (N = 136)	123 (90.4)	Ref	Ref
Copy of treatment guidelines or reference (N = 411)	379 (92.2)	1.1 (0.5-2.6)	0.6 (0.2-1.4)
No copy of guideline (N = 92)	84 (91.3)	Ref	Ref
**Patient seen by health worker who had:**			
Supervision in previous six months(N = 110)	100 (91.0)	0.8 (0.3-1.9)	0.6(0.3-1.0)
No supervision previous six months (N = 393)	363 (92.4)	Ref	Ref
Training on use of artemether-lumefantrine (N = 224)	203 (90.6)	0.7 (0.4-1.3)	0.5 (0.2-1.2)
No training on use of artemether-lumefantrine (N = 279)	260 (93.2)	Ref	Ref
**Patient age:**			
<3 years (N = 177)	172 (97.2)	Ref	Ref*
3- <9 years (N = 145)	123 (84.8)	0.1 (0.05-0.5)	0.1 (0.04-0.4)
9- <12 years (N = 33)	27 (81.8)	0.1(0.04-0.4)	0.1 (0.03-0.3)
12 years and above (N = 148)	141 (95.3)	0.6 (0.2-1.9)	0.6 (0.2-2.2)
**Patient weight:**			
5 -15 kg (N = 241)	234 (97.1)	Ref	Ref*
>15 -25 kg (N = 88)	70 (79.5)	0.1 (0.04-0.3)	0.1 (0.03-0.3)
>25- 35 kg (N = 24)	18 (75.0)	0.1 (0.03-0.2)	0.06 (0.01-0.2)
>35 kg (N = 150)	141 (94.0)	0.6 (0.2-2.1)	0.8 (0.2-2.8)
**Other characteristics:**			
Had fever with present illness (N = 463)	449 (92.0)	1.2 (0.2-6.5)	0.6 (0.07-4.5)
No fever this illness (N = 15)	14 (93.2)	Ref	Ref

*Due to collinearity of age and weight, multivariate models did not include both age and weight in a single model.

Table 5 describes the relationship between patients’ age and weight based on AL dosing criteria. The findings indicate that the weight for age are not concordant for the two middle weight groups (15-25 kg and >25-35 kg). There are patients who weigh less or more than expected compared to their age group. For example, a total of 144 patient aged three to nine years were expected to receive two AL tablets corresponding to weight band 15- < 25 kg, but only 73 (50.7%) fell in this weight band; 67 (46.5%) had low weight for age and four (2.8%) weighed too much for their age.

**Table 5:** Relationship between weight and age of patients who received artemether-lumefantrine based on recommended artemether-lumefantrine dosing bands (N = 503)

**Dosing criteria**	**Age <3 years**	**3 - <9 years**	**9 t - < 12 years**	**12 years and above**	**Total**
<15 kg	175 (98.8%)	67 (46.5%)	2 (6.1%)	1 (0.7%)	245 (48.7%)
15-25 kg	2 (0.2%)	73 (50.7%)	14 (42.4%)	-	89 (17.7%)
>25-35 kg	-	4 (2.8%)	14 (42.4%)	6 (4.0%)	24 (4.8%)
>35 kg	-	-	3 (9.1%)	142 (95.3%)	145 (28.8%)
Total	177 (100%)	144 (100%)	33 (100%)	149 (100%)	503 (100%)

## Discussion

This study assessed the association of health facility and patient characteristics to correct AL dosing for the treatment of uncomplicated malaria in rural Tanzania. Malaria treatment guidelines in Tanzania follow manufacturers’ recommendations, which allow for both age- and weight-based AL dosing [3]. More than four years after the introduction of AL, correct AL dosing was suboptimal in some patient groups, especially children aged three to 12 years. Other health facility and patient factors were not associated with correct dosing.

Concerns about incorrect dosing of anti-malarial drugs have been reported in other settings [6,7,14]. In particular, a study from Kenya showed that infants were more likely to receive appropriate treatment than older children [11]; the authors suggested that health workers were being more careful with the younger age group, which seems a logical explanation for the observation. In this study, 35.2% of all patients receiving AL were children aged three to 12 years, many of whom did not receive correct AL dosing. Correct AL dosing is particularly important for children as they are more likely to contract malaria, more likely to progress to severe illness, and more likely to die from malaria than adults.

Although, 92.1% of patients received correct AL dosing by age or weight criteria, fewer received correct dosing by weight-based criteria alone (85.7%) or age-based criteria alone (78.5%). Our analysis of weight versus age profile of this patient population suggests that the weight-to-age profile needs to be adapted to the local population context. Although, the weight-to-age profile matches for children <3 kg (98.8% concordant with age) and persons >35 kg (95.3% concordant with age), the weight-to-age profile does not match for children 15 to <25 kg (50.7% concordant) and for children 25 to <35 kg (42.4% concordant); most of the dosing errors based on age alone would lead to overdosing compared to dosing based on weight alone. In addition, weight-based prescriptions should be better implemented to ensure appropriate dosing. In this study, almost all (95.8%) of patients had their age assessed, but only 33.8% of patients had their weight assessed. Thus, to improve weight-based dosing health workers should be encouraged to assess weight in all patients.

AL is supplied to health facilities in four different dose packs. An informal observation during the survey found that in the event that a correct AL dose pack for the appropriate age or weight group was not in stock, the provider would use any AL package to compensate for the missing package. For example, an 11 year old who needs three AL tablets dose (pink package), may receive two packs: one blue pack (toddler package with two AL tablets per dose) and one yellow pack (baby package with one tablet per dose). This strategy might not always work well and might confuse the patient about the appropriate number of pills required. More assessment of this phenomenon is needed to understand its effect in providing adequate care for malaria patients. In addition, we do not know the impact of uniform AL packaging (e.g. adults only, or loose tablets) on correct AL dosing.

During treatment policy change it is customary to train health workers on new guidelines and provide reference materials for use upon returning to the health facility. In this assessment, neither health workers’ training on AL use nor possession of reference material improved the odds of correct AL dosing. One explanation for this finding could be that it takes time and experience for trainees to be competent in new topics. This underscores the need for frequent supervision from health management teams, with possibilities of refresher training and/or on-the-job training, to complement formal training. Moreover, training content, modality, and duration could influence providers’ understanding and performance post-training; all of which were not assessed in this study.

Receipt of supervisory visits was not associated with correct dosing, and very few patients (21.8%) were seen by providers who have had a supervisory visit in the previous six months. The study did not assess the type or content of supervision, which might be important factors. The role of supervision visits in improving quality of malaria case management has provided inconsistent conclusions in other settings [11]. However, understanding predictors of appropriate care for malaria patients can assist health managers in planning resources and performing supportive supervision with emphasis on areas seem to be challenging, in order to improve the quality of services and support disease control measures.

### Limitations

No information was collected on total number of patients attended by a provider to assess caseload and how it may have affected quality of care, but all patients and providers on the day of survey were included, hence the patient sample is self-weighting on the basis of utilization for the days surveyed, assuming survey days were typical for the rest of the year. The importance of caseload assessment has been described elsewhere [6,11,15].

## Conclusions

Correct AL dosing in the study areas was generally high, but children aged three to 12 years were significantly less likely to receive correct AL dosing. Health workers should be made aware of the possibility of incorrect dosing for older children and young adults in the middle age and weight categories of the current AL formulation in use. Supportive measures to ensure availability of basic equipment and an emphasis on weight-based AL dosing should be made to improve clinical practise.

## Competing interests

The study was carried as part of the phase 4 platform on systems effectiveness and safety of ACT in Africa (INESS). The INESS project was primarily funded by the Bill and Melinda Gated Foundation through a sub-grant to the INDEPTH Network. The authors have declared that they have no competing interests.

## Authors’ contributions

MIM trained and supervised field workers, analysis, prepared the first draft; MS and DK assisted in training of field workers, performed statistical analysis, revision of first and subsequent drafts; RAK and BA supervised field work and revision of draft manuscripts; IK, DdS, SPK and JS: study design, technical support and revision of subsequent manuscripts. All authors read and approved the final manuscript.
